# Role of EmaSR in Ethanol Metabolism by *Acinetobacter baumannii*

**DOI:** 10.3390/ijms232012606

**Published:** 2022-10-20

**Authors:** Hung-Yu Shu, Yu-Wen Huang, Ping-Yi Tsai, Kun-Sheng Hsieh, Guang-Huey Lin

**Affiliations:** 1 Department of Bioscience Technology, Chang Jung Christian University, Tainan 711301, Taiwan; 2 Department of Life Sciences, School of Medicine, Tzu Chi University, Hualien 970374, Taiwan; 3 Department of Microbiology and Immunology, School of Medicine, Tzu Chi University, Hualien 970374, Taiwan

**Keywords:** *Acinetobacter baumannii*, two-component system, alcohol metabolism, acetate metabolism, bacterial motility, biofilm formation, virulence, resistance

## Abstract

*Acinetobacter baumannii* is a well-known nosocomial pathogen that can survive in different environments through the use of intricate networks to regulate gene expression. Two-component systems (TCS) form an important part of such regulatory networks, and in this study, we describe the identification and characterization of a novel EmaSR TCS in *A. baumannii*. We constructed a Tn*5*-tagged mutagenesis library, from which an *emaS* sensor kinase gene and *emaR* response regulator gene were identified. We found that *emaS*/*emaR* single-mutants and double-mutants were unable to replicate in M9 medium with 1% ethanol as the single carbon source. Motility and biofilm formation were negatively affected in double-mutants, and transcriptomic analysis showed that mutation of *emaSR* dysregulated genes required for carbon metabolism. In addition, *emaS*/*emaR* single-mutants and double-mutants were unable to survive in acetic acid- and sodium acetate-containing medium, indicating that the EmaSR TCS is also important for acetate metabolism. Furthermore, virulence against *Galleria mellonella* was diminished in *emaS*/*emaR* single- and double-mutants. Taken together, these results show that this novel EmaSR TCS is involved in the regulation of *A. baumannii* ethanol metabolism and acetate metabolism, with important implications on motility, biofilm formation, and virulence if mutated. Further research on the underlying mechanisms is warranted.

## 1. Introduction

*Acinetobacter baumannii* is an emerging Gram-negative nosocomial pathogen that is a rising cause of hospital-acquired infections [1], and which has an extraordinary ability to acquire antibiotic resistance factors [2]. In addition to conventional antibiotic resistance genes, previous research has shown that genes involved in *A. baumannii* indole-3-acetic acid biosynthesis and metabolism [3] and ethanol metabolism [4] can also play a role in stress resistance and virulence. Two-component systems (TCSs) also play an important part in *A. baumannii* stress resistance, biofilm formation, and drug resistance, primarily by regulating the signal transduction of environmental stimuli. At least 7 TCSs have been described in *A. baumannii* thus far*,* including AdeSR [5], BaeSR [6], BfmSR [7], GasSA [8], PmrAB [9], StkSR [10], and A1S_2811 of *A. baumannii* ATCC 17978 [11], and have been associated with aromatic compound catabolism, motility, biofilm formation, virulence, and drug resistance [12]. 

In our previous research, in silico analysis of *A. baumannii* revealed at least 7 genes that were annotated as alcohol dehydrogenases (Adhs), of which three iron-containing Adhs were selected for detailed study. Markerless-mutation revealed that Adh4 exhibited the strongest enzymatic activity toward ethanol [4]. To better understand gene regulation of ethanol metabolism in *A. baumanii*, a Tn*5*-tagged mutagenesis library was constructed. A mutant with a transposon inserted in the *DJ41_3172* gene failed to retain the ability to survive with ethanol as the sole carbon source. This gene was annotated as a sensor kinase, and a response regulator, *DJ41_30170*, was subsequently identified upstream of the *DJ41_3172* gene. Together, *DJ41_3172* and *DJ41_3170* constitute a TCS involved in *A. baumannii* ethanol metabolism, and thus we termed these genes as *emaS* (ethanol metabolism a sensor) and *emaR* (regulator), respectively. In this study, we sought to further elucidate the role of this novel TCS in *A. baumannii* ethanol metabolism, motility, biofilm formation, stress resistance, and virulence. 

## 2. Results

### 2.1. The EmaSR TCS in A. baumannii

**Figure 1:**
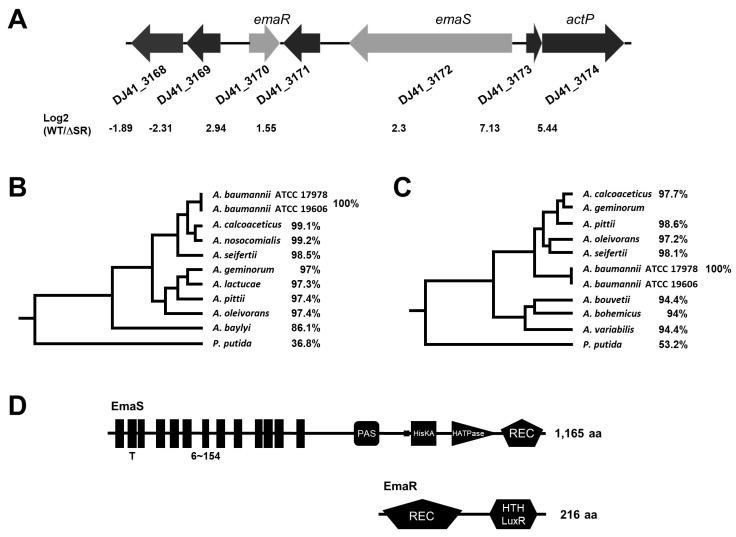
Gene arrangement and phylogenetic comparison of *emaS* and *emaR*. (**A**) Arrangement of *emaS* and *emaR* and surrounding genes (spanning *DJ41_3168*~*DJ41_3174*). The numbers at bottom indicate the relative gene expression ratio of wild-type to *emaSR* (ΔSR) mutant. (**B**) Phylogenetic trees constructed from amino acid sequence alignments of EmaS-like proteins from *Acinetobactor* species and MxtR of *P. putida.* Percentages indicate the amino acid identity of each protein to EmaS of *A. baumannii*. (**C**) Phylogenetic trees constructed from amino acid sequence alignments of EmaR-like proteins from *Acinetobactor* species and ErdR of *P. putida.* Percentages indicate the amino acid identity of each protein to EmaR of *A. baumannii*. (**D**) Domain structure of EmaS and EmaR. T, transmembrane domain; PSA, Per-Arnt-Sim domain; HisKA, histidine kinase domain for phosphorylation; HATPase, histidine ATPase domain; REC, response regulator domain; HTH LuxR, LuxR family helix-turn-helix domain.

To date, 7 alcohol dehydrogenases (Adhs) have been annotated in the genome of *A. baumannii.* We previously studied the 3 iron-containing Adhs among these, and found that Adh4 is responsible for alcohol degradation, while Adh3 and Adh6 are involved in stress resistance [4]. To better understand the signal transduction of alcohol metabolism-related genes, a Tn*5*-mediated transposon mutant library was constructed, and screening for genes with loss of function for alcohol metabolism was conducted in *A. baumannii* ATCC 19606. A mutant that lost the ability to survive in medium containing ethanol as the sole carbon source was identified, and sequencing analysis revealed a Tn*5* insertion in the 1823-base pair (bp) *DJ41_3172* sensor kinase gene. A putative response regulator gene, *DJ41_3170*, was subsequently discovered upstream of the *DJ41_3172* gene (Figure 1A). Together, the *DJ41_3172* and *DJ41_3170* genes bear the features of a potential TCS, and were therefore respectively named as *emaS* (ethanol metabolism a sensor) and *emaR* (regulator).

Phylogenetic cladograms were developed, using available genomic information from *A. baumannii* ATCC 19606, 9 other different *Acinetobacter* species, and *Pseudomonas putida* (Figure 1B,C). These 9 other *Acinetobacter* species and *P. putida* are known to be opportunistic human pathogens, and can cause disease when suitable conditions arise. Although cases of lethal infections in elderly or immunocompromised individuals have been reported in the literature, clinical knowledge remains limited overall; however, cell culture and animal studies are endeavoring to shed light on the relationships between gene expression and pathogenicity or virulence in these species. For most *Acinetobacter* species, EmaS shared more than 97.1% amino acid identity with similar enzymes identified, except for *A. baylyi*, where only 86.1% similarity was observed (Figure 1B). Interestingly, EmaS shared 36.8% identity with MxtR, a sensor kinase essential for acetate utilization in *P. putida* [13]. Amino acid alignment revealed >94% identity with similar proteins in all assessed *Acinetobacter* species for EmaR, and 53.2% identity with ErdR, a cognate response regulator of MxtR in *P. putida* KT2440, was also noted (Figure 1C). In addition, SMART (http://smart.embl-heidelberg.de/ accessed on 16 October 2022) analysis revealed a potential sensor input domain with 13 transmembrane regions (T), spanning amino acids (aa) 6 to 154 in EmaS, as well as Per-Arnt-Sim (PAS) and His Kinase A (HisKA) domains, an HATPase for autophosporylation, and a REC receiver domain for response regulation (Figure 1D). This domain structure was similar to *P. putida* MxtR. SMART analysis of EmaR revealed a REC domain that can receive phosphate from the sensor kinase, as well as a LuxR-like helix-turn-helix (HTH) domain for DNA binding that shared high similarity with *P. putida* ErdR (Figure 1D). 

### 2.2. EmaSR Functions in Ethanol Metabolism

To elucidate the function of EmaSR, knockout strains were constructed using allelic exchange as previously described [14], and *emaS* (ΔS) and *emaR* (ΔR) single-mutants, as well as *emaSR* (ΔSR) double-mutants, were generated. Complementary plasmids with the promoter of each gene were constructed in pWH1266 to generate pS, pR and pSR (Table 1). Mutants with the *emaS* deletion were checked by polymerase chain reaction (PCR) using the emaS-eR and emaS-eF primers (Figure S1A). Results revealed that no PCR product was detected in *emaS* mutants (Figure S1B, lane 5, 7, 11 and 13). Complementary strains with pR and pS were checked using primers emaR-eF and emaS-eF, respectively, in tandem with the Tc-R primer (Figure S2A). PCR results confirmed the accuracy of the complementary strains (Figure S2B).

**Figure 2:**
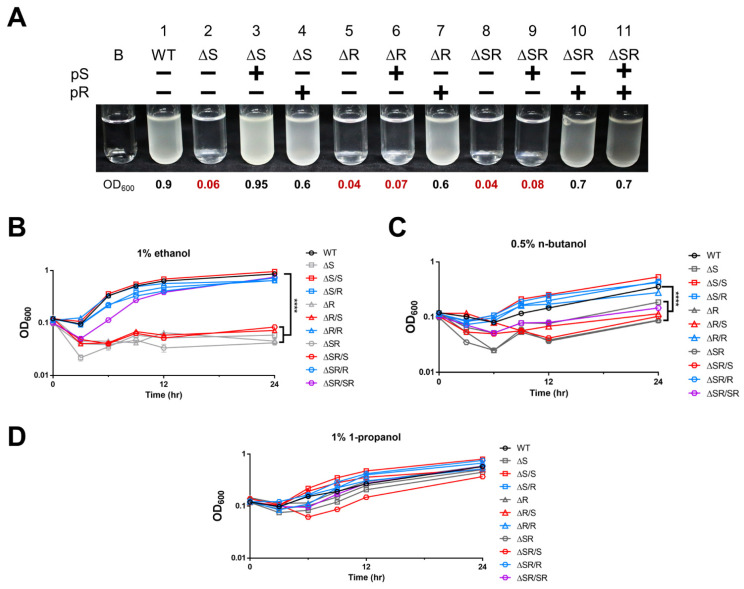
Growth conditions of mutant and complementary strains in M9 medium containing alcohols. (**A**) Strains cultured in test tubes for 24 hours. B stands for “blank,” and contains M9 medium only. WT represents wild-type. Numbers under the test tubes represent OD at 24 hours. Growth curves of strains cultured in M9 medium with (**B**) 1% ethanol (B), (**C**) 0.5% n-butanol, and (**D**) 1% 1-propanol are shown. Gray lines represent single- or double-mutants, while red lines indicate mutants complemented with pS, blue lines indicate mutants complemented with pR, and purple lines indicate double-mutants complemented with pSR. Representative results from three independent experiments are shown. **** *p* < 0.0001 (WT vs. ΔS, ΔR, ΔR/S, ΔSR, ΔSR/S).

Mutant and complementary strains were cultured in LB media containing relevant antibiotics with agitation overnight. Overnight cultures were then washed twice with M9 medium and resuspended in M9 medium containing 1% ethanol, 1% 1-propanol, or 0.5% n-butanol with 0.1 of initiate optical density (OD). After culturing for 12 hours, results showed that ΔS, ΔR, and ΔSR mutants were unable to survive in M9 medium containing 1% ethanol (Figure 2A, lane 2, 5, 8), while pS complementary strains restored survival for ΔS but not the ΔSR double mutant (Figure 2A, lane 3, 9). Interestingly, pR was able to restore survival not only for ΔR, but also ΔS and ΔSR. This indicates that overexpression of EmaR can complement the dysfunction of EmaS in the ΔS and ΔSR mutants (Figure 2A, lane 4, 10). The OD of each strain was determined at different timepoints until the 24th hour. Results indicated that mutants which lost the ability to survive remained at low density over 24 hours (Figure 2B, gray lines). All mutant strains with pR demonstrated growth (Figure 2B, blue lines), but only ΔS strains with pS showed growth (Figure 2B, red lines). Together, the results show that EmaS is essential for ethanol metabolism, but EmaR may help to enhance survival as a cognate response regulator. The survival of mutant and complementary strains in other alcohols was also tested, and similar results were observed for cultures in M9 medium containing 0.5% n-butanol (Figure 2C). However, all strains were able to survive in M9 medium containing 1% 1-propanol (Figure 2D), with no significant difference between strains, and this suggests that the EmaSR system is not essential for the regulation of 1-propanol metabolism. 

### 2.3. EmaR Serves as the Cognate Regulator of EmaS

**Figure 3:**
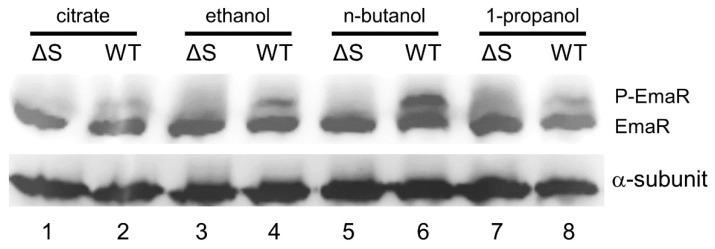
Phos-tag^TM^ analysis of P-EmaR. *A. baumannii* wild-type and ΔS mutant strains were cultured in M9 medium containing 5 mM citrate (lane 1, 2), 5 mM citrate with 0.5% ethanol (lane 3, 4), 5 mM citrate with 0.5% n-butanol (lane 5, 6), or 5 mM citrate with 0.5% 1-propanol. The anti-α subunit was used as an internal control.

Complementation of pR restored ethanol metabolism in the ΔS single mutant and ΔSR double mutant, indicating that overexpressing EmaR by transforming pR into the ΔS or ΔSR mutants can compensate for EmaS deficiencies. As auto-phosphorylation of sensor proteins and phosphate transfer to the cognate regulator is important evidence for a TCS, we conducted Phos-tag^TM^ electrophoresis with wild-type and ΔS strains to understand the phosphorylation dynamics between EmaS and EmaR. Wild-type and ΔS strains were cultured in M9 medium containing ethanol, n-butanol, or 1-propanol. Cultures were then lysed, and proteins prepared for electrophoresis on a 12% sodium dodecyl sulfate (SDS)-acrylamide gel containing 50 μM Phos-tag^TM^. Western blotting with anti-EmaR antibody revealed that phosphorylated EmaR (P-EmaR) was not detectable in the ΔS mutant (Figure 3, lane 1, 3, 5, 7). Interestingly, the P-EmaR band had similar intensity to non-phosphorylated EmaR in wild-type strains cultured in M9 medium containing n-butanol (Figure 3, lane 4). However, the intensity of the P-EmaR band was just 20% of the non-phosphorylated EmaR band in wild-type strains cultured in M9 medium that contained 1% ethanol and 1% 1-propanol (Figure 3, Lane 4, 8). Phos-tag^TM^-electrophoresis demonstrated that EmaR phosphorylation was induced not only by ethanol, but also by n-butanol and 1-propanol. Reduction of P-EmaR in the ΔS mutant provided additional evidence that EmaR serves as the cognate response regulator of EmaS. 

### 2.4. Loss of EmaSR Increased Oxidative Resistance but Reduced Motility and Biofilm Formation Ability

**Figure 4:**
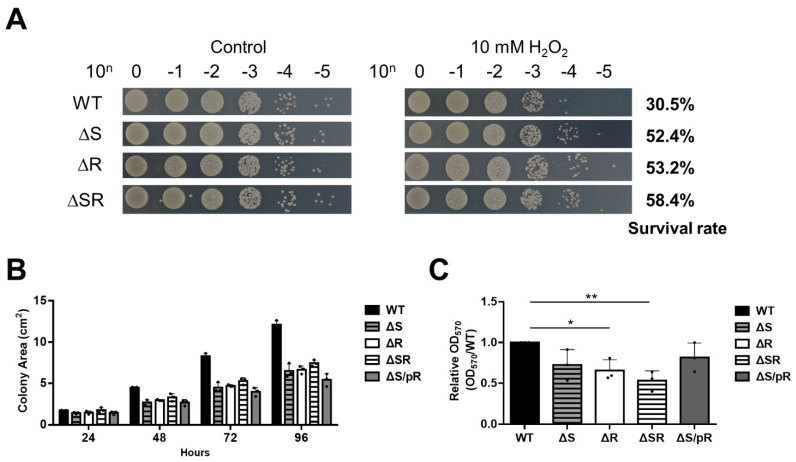
Oxidative resistance, motility, and biofilm formation capabilities in EmaSR mutant strains. (**A**) Inorganic oxidative resistance assay results. Bacteria were cultured until OD_600_ of 0.6, and treated with 10 mM H_2_O_2_ for 20 minutes. Five μL each of bacteria were dropped onto LB agar plates to determine the survival rate. (**B**) Motility assay results. Each strain was cultured in 0.25% LB agar for 72 hours. Swimming areas evaluated in triplicate for each strain was determined using ImageJ at the time points indicated. (**C**) Quantification of biofilm formation in polystyrene 96-well plates. Relative OD_570_ was determined after crystal violet staining. Experiments were performed in triplicate. * *p* < 0.05; ** *p* < 0.01.

Previous studies indicate that ethanol can trigger stress resistance mechanisms in *A. baumannii* [4,18], and as the EmaSR system was shown to be associated with ethanol metabolism, we sought to understand whether EmaSR also had a role in stress resistance. Accordingly, we treated wild-type and mutant strains with 10 mM H_2_O_2_ for 20 min, and the results showed that the ΔS, ΔR, and ΔSR mutants had increases in survival rate of at least 20% in comparison with wild-type (Figure 4A).

In addition, we performed a motility assay with wild-type and mutant strains on 0.5% agar LB plates. Migration areas were quantified using ImageJ, and results indicated that mutants lost motility, with migration areas reduced by 2-fold in comparison with wild-type. Interestingly, pR did not restore ΔS motility under these circumstances (Figure 4B, gray bar). 

The biofilm forming ability of wild-type and mutant strains was also assessed, in cultures with OD of 0.1 that were grown in LB medium for 12 hours. All mutants showed declines in biofilm-forming ability, with the ΔR single-mutant and ΔSR double-mutant demonstrating a significant difference in comparison with wild-type. However, pR was able to restore most of the biofilm-forming ability of the ΔS mutant (Figure 4C, gray bar). These results show that the EmaSR system can also play a role in motility and biofilm formation to enhance stress resistance.

### 2.5. Gene Expression Changes Respectively Caused by Knockout of EmaSR, EmaS, and EmaR

**Figure 5:**
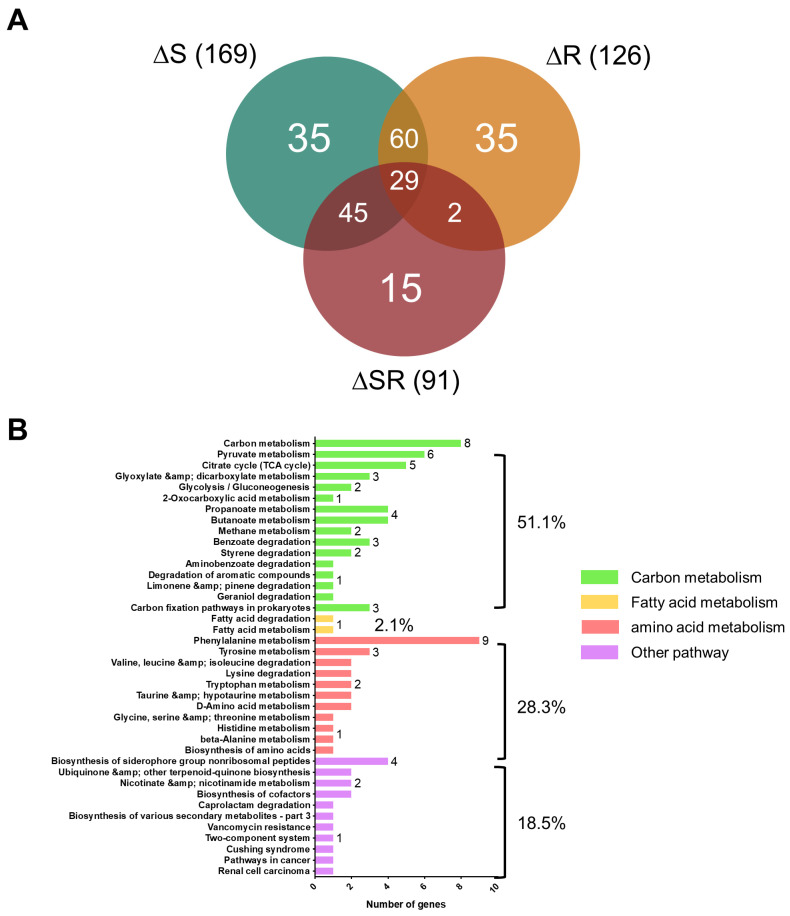

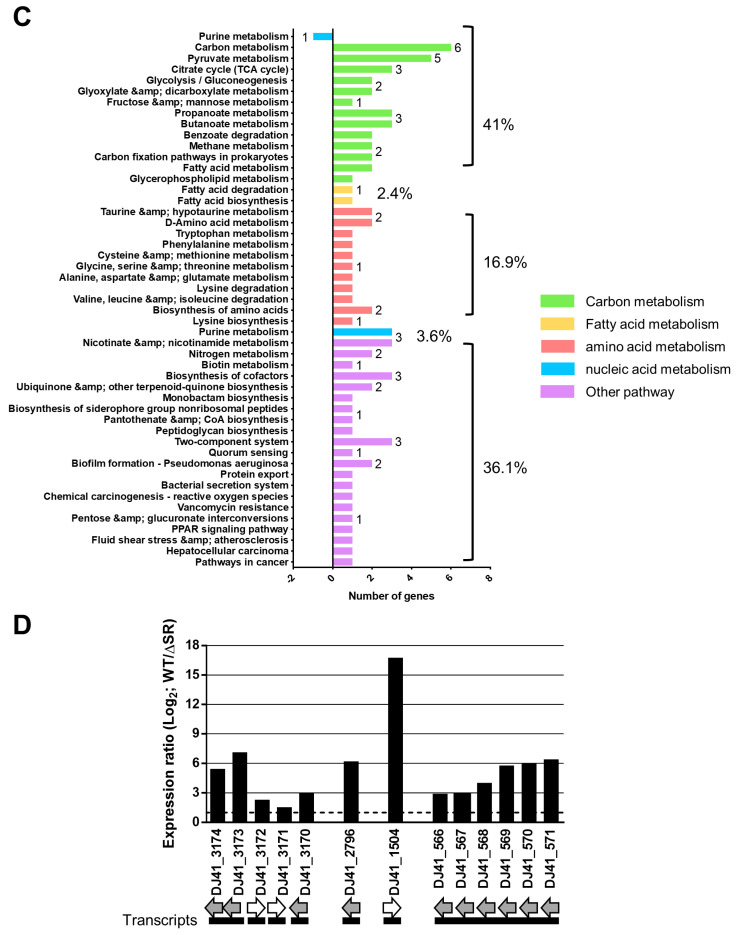
Transcriptome analysis of differential gene expression between *A. baumannii* wild-type and the *emaSR*, *emaR*, or *emaS* mutant strains. (**A**) Total number of upregulated genes for the *emaS*, *emaR*, and *emaSR* mutant strains in comparison with wild-type. (**B**) Metabolic classification of differentially expressed genes activated by EmaSR. (**C**) Metabolic classification of differentially expressed genes regulated by EmaR. Only one gene involved in purine metabolism was repressed by EmaR (blue bar at top). (**D**) Gene expression ratios of two highly expressed gene clusters. Black lines indicate individual transcriptional units. The dashed line represents the average expression ratio for all gene clusters depicted in each panel for one representative experiment.

RNA sequencing was performed in wild-type and mutant strains cultured in 5 mM citrate containing M9 medium supplemented with 0.5% ethanol for three hours. Bacteria were collected and sent to Welgene Biotech for RNA sequencing analysis. A total of 169, 126, and 91 genes respectively demonstrated differential expression activity for the ΔS, ΔR, and ΔSR mutant strains in comparison with wild-type (Figure 5A). The number of genes regulated by ΔSR was fewer than that found in ΔS or ΔR (91 vs. 169 vs. 126 genes), indicating that EmaS or EmaR might cooperate with other TCS for signal transduction in *A. baumannii*. Most genes were found to be positively regulated by EmaSR. Only 50 genes were found to be negatively regulated by EmaSR (data not shown). From a total of at least 3750 annotated open reading frames (ORFs), 386 genes showed differential expression patterns. Results demonstrated that following mutation of *emaSR*, expression of 91 genes was significantly upregulated (log2.Fold_change > 2; Table S1); in addition, 126 genes were significantly upregulated in the *emaR* mutant (log2.Fold_change > 1; Table S2), and 169 genes were significantly upregulated in the *emaS* mutant (log2.Fold_change > 1; Table S3). For the 91 EmaSR upregulated genes, 51.1%, 28.3%, and 36.1% were respectively involved in carbon metabolism, amino acid metabolism, and other pathways (Figure 5B). Upregulated genes were enriched in the KEGG pathway of ethanol metabolism, including acetyl-CoA and other substrates involved in the TCA cycle. In analyzing highly differential gene clusters of the EmaSR and EmaR upregulated transcriptomes, *DJ41_3173*, *DJ41_3174* and the neighboring genes of EmaSR encoded genes were observed to have reduced expression levels in the ΔSR mutant (Figure 5D). Another gene cluster that demonstrated elevated expression levels from 2.9~6.42 of log_2_ ratio, *DJ41_571*~*DJ41_566*, was discovered to be involved in acetoin catabolism (Figure 5D).

**Figure 6:**
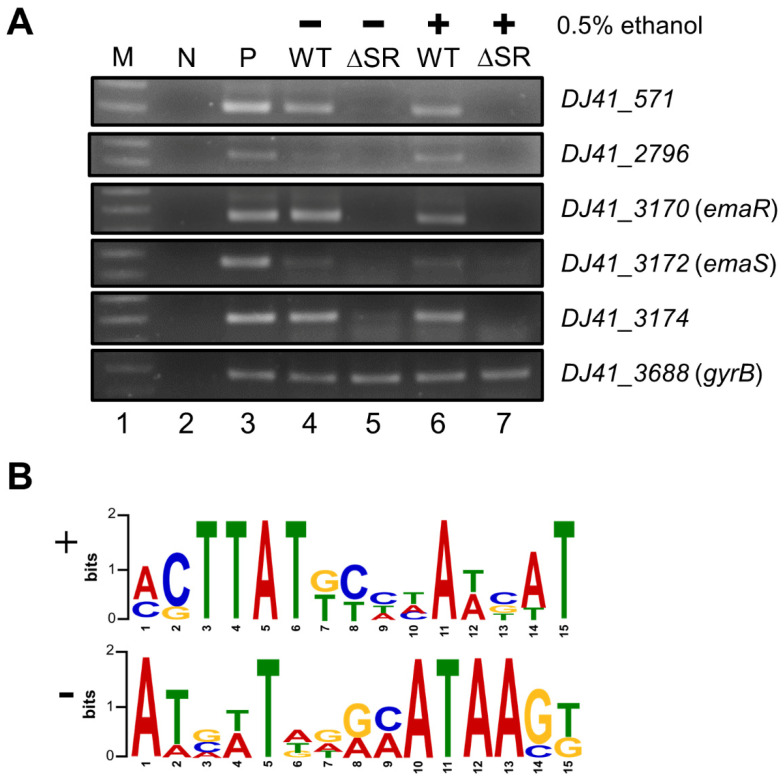
Transcriptional expression analysis and putative EmaR binding box analysis. (**A**) RNA expression analysis by RT-PCR of genes upregulated by EmaSR. Lane 1 is a marker, and lane 2 is non-template DNA used as a negative control. Lane 3 is a positive control with DNA as template. Lanes 4 and 6 contain RNA extracted from wild-type strains cultured without or with 0.5% ethanol. Lanes 5 and 7 contain RNA extracted from the *emaSR* mutant strain cultured without or with 0.5% ethanol. *DJ41_3688* is a gyrase-encoding gene that served as an internal control. (**B**) Putative EmaR binding box analysis by MEME. The upper panel is the highly conserved 15 bp-sequence on the positive strand (+). The lower panel is the highly conserved 15 bp-sequence on the negative strand (-).

TCS regulate gene expression in response to different stimuli, and have also been reported to self-regulate expression [12]. Reverse transcription was performed to confirm the expression of regulated genes in wild-type and ΔSR mutant strains when cultured in the presence or absence of ethanol. Gene expression was highly induced in *DJ41_571*, *DJ41_2796*, *DJ41_3174*, *emaR*, and *emaS*, and these results were further verified by RT-PCR and found to correlate well with the RNA sequencing data. The results of RT-PCR agreed with the transcriptomic analysis, which suggested that EmaSR activated neighboring genes and themselves in the presence of ethanol (Figure 6A, lane 5, 7).

In order to locate possible EmaR binding sites, we conducted MEME analysis (https://meme-suite.org/meme/tools/meme accessed on 16 October 2022) to identify regulatory regions within differentially expressed genes. The EmaR binding motif is located within a 15 bp-region, with a sequence of 5`xCTTATxxxxAT(A)xAT3` on the positive strand (Figure 6B, upper panel). These sequences were also found to be consistent with those located in the upstream region of genes with opposite transcriptional orientation (Figure 6B, lower panel). The EmaR binding motif was also found in the upstream regions of *emaS* and *emaR*, indicating that EmaR can regulate TCS gene expression (Table 2), and that EmaSR can also self-regulate transcription, which is consistent with the features of most TCS.

**Figure 7:**
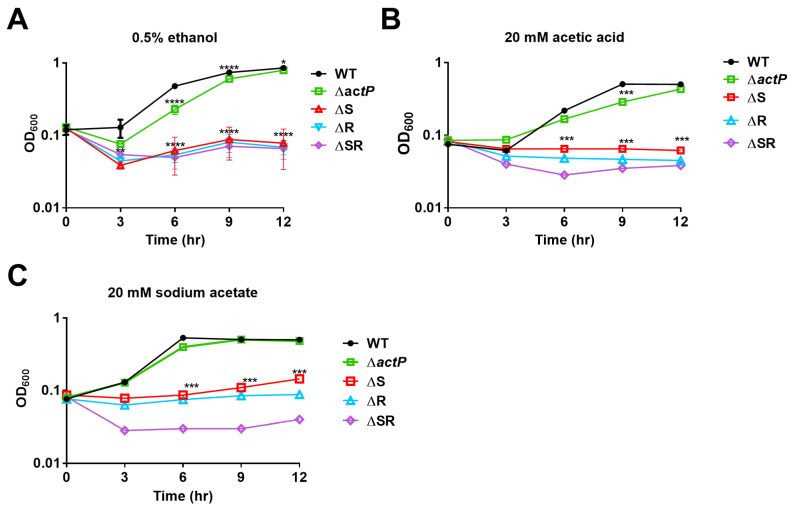
Growth curves of strains in (**A**) 0.5% ethanol, (**B**) 20 mM acetic acid, and (**C**) 20 mM sodium acetate. Black, green, red, blue, and purple lines respectively indicate wild-type and the *actP*, *emaS*, *emaR*, and *emaSR* mutants, respectively. Representative results from three independent experiments are shown. * *p* < 0.05; *** *p* < 0.001; **** *p* < 0.0001.

Transcriptome data demonstrated that *DJ41_3173* and *DJ41_3174* in the neighboring regions of *emaSR* were upregulated by EmaSR (Table S1, Figure 1A). *DJ41 _3174* has been annotated as having solute transporter function for the sodium symporter family protein, *actP.* In order to further elucidate the function of *actP*, a knock-out mutant was generated by allelic exchange with homologous recombination, using methods that were previously described [4]. Growth conditions for *ΔactP*, as well as the *emaS* and *emaR* single- and double-mutants, were observed while strains were cultured in M9 medium containing 0.5% ethanol, 20 mM acetic acid, or 20 mM sodium acetate. Results revealed that *ΔactP* had reduced growth rates when cultured in 0.5% ethanol during the first 6 hours, and then reached similar growth rates as wild-type after the 9th hour. Meanwhile, the *emaS* and *emaR* single- and double-mutants had the same optical density even after culturing for 12 hours (Figure 7A). When cultured in 20 mM acetic acid, the *ΔactP* mutant had low OD of 0.28 at 9 hours, but reached the same level as wild-type after culturing for 12 hours. However, the *emaS* and *emaR* single and double mutants lost growth ability and remained in the same condition even after culturing for 12 hours. (Figure 7B). When cultured in 20 mM sodium acetate for 12 hours, *ΔactP* showed the same growth rates as wild-type, but the *emaS* and *emaR* single- and double-mutants also lost growth ability (Figure 7C). These results demonstrate that EmaSR likely regulates ethanol metabolism and also acetate metabolism. However, although *actP* gene expression can be regulated by EmaSR (Table S1, Figure 1A), the *actP* mutant retained the ability to survive in acetic acid- or sodium acetate-only medium, and this indicates that *A. baumannii* likely has other acetate metabolism genes to complement the loss of *actP*.

### 2.6. EmaS and EmaR Are Associated with Virulence against G. mellonella

**Figure 8:**
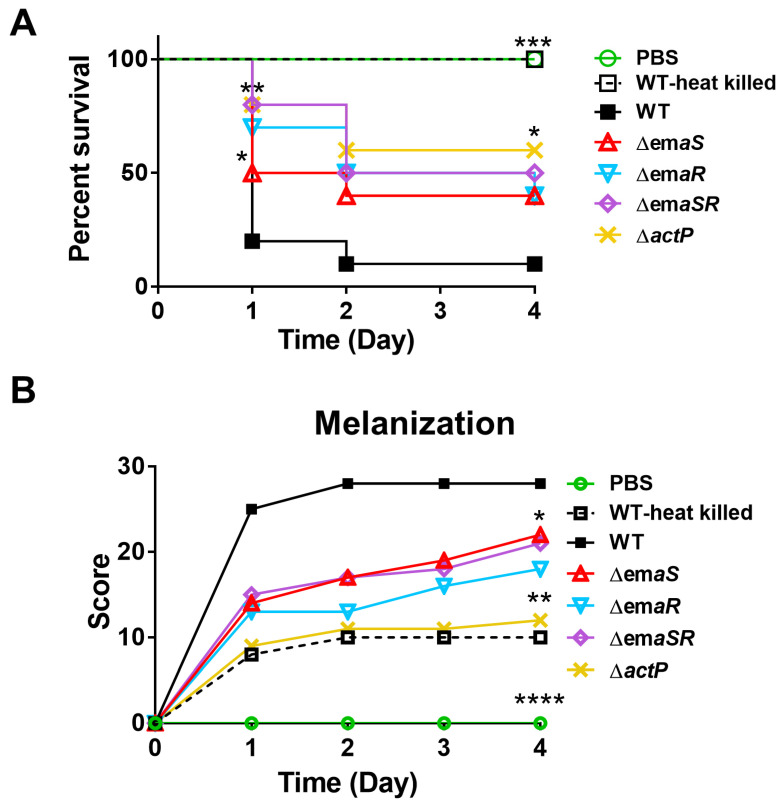
*G. mellonella* survival rates after infection with *A. baumannii* and mutant strains. (**A**) Kaplan-Meier survival curves, with each curve representing a single representative experiment performed with 10 larvae. (**B**) Melanization score curves, with each curve representing a single representative experiment performed with 10 larvae. * *p* < 0.05; ** *p* < 0.01; *** *p* < 0.001; **** *p* < 0.0001.

Ethanol-induced stress resistance has been associated with higher virulence in our previous research [4], and to examine if there was a correlation between virulence and EmaSR function, *G. mellonella* model organisms were used. Ten larvae for each group were prepared, and respectively injected with 5 × 10^6^ colony-forming units (CFU) of different wild-type or mutant strains. Survival curves were recorded each day for 4 days. Kaplan-Meier survival curves showed that 9 larvae injected with the wild-type strain died within 24 h after injection, while all 10 larvae injected with either phosphate-buffered saline (PBS) or a heat-killed wild-type strain survived. Larvae injected with *emaS*, *emaR*, *emaSR*, and *actP* mutants maintained around 50% survival rate at 96 hours post-injection (Figure 8A). These observations suggest that EmaSR can regulate virulence genes, and ActP also contributes to virulence against *G. mellonella*. Melanization status was scored as previously described [4], and the highest melanization scores were seen in larvae injected with the wild-type strain, which agrees with the poor survival rate observed for this group. However, larvae injected with other mutants presented similar levels of melanization as the group injected with the heat-killed wild-type strain (Figure 8B).

**Figure 9:**
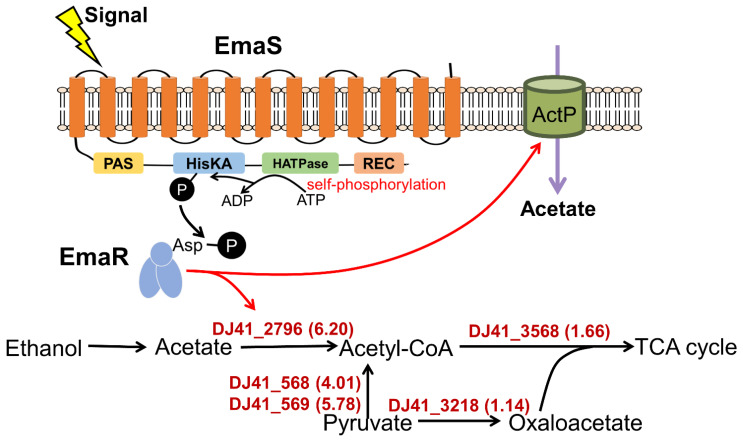
Schematic outlining the role of the EmaSR system in relation to the metabolic network of *A. baumannii*.

Taken together, a novel TCS EmaSR was found and studied in this article. Deletion of EmaSR resulted in loss of survival in medium containing 1% ethanol as the sole carbon source. EmaR serves as the cognate response regulator of EmaS, as indicated by pR complementation of the ΔS mutation and Phos-tag^TM^ electrophoresis results. Through RNA sequencing results, EmaSR was shown to regulate genes in neighboring regions, as well as self-regulate. EmaSR also possessed the ability to regulate genes involved in acetate and acetoin catabolism. The relationship between the EmaSR TCS and the metabolic network in *A. baumannii* is depicted in Figure 9.

## 3. Discussion

Most TCS found in bacteria to date have the ability to regulate gene expression for antibiotic resistance [10,19], antibiotic production [20], virulence, biofilm formation and motility [21]. In addition, an increasing number of studies show that TCS are involved in metabolism; for example, the MxtR/ErdR TCS has been shown to be essential for acetate utilization in *P. putida* KT2440 [13]. Although none of the three predicted *actP* genes of *P. putida* KT2440 were upregulated by acetate, the band shift of the fragment containing the promoter of the *actP-I* operon indicates binding of ErdR to this region. *DJ41_3174* has been annotated as an ActP coding gene, and was upregulated (WT/ΔSR = 5.44) by EmaSR (Table S1) in *A. baumannii* (Figure 1A). However, the *actP* mutant of *A. baumannii* did not lose the ability to survive in 20 mM acetate or sodium acetate after culturing for at least 12 hours (Figure 7B,C), indicating that there may be other acetate metabolism genes that can complement *actP*. Other examples of TCS involved in metabolism include TCS09 of *Streptococcus pneumoniae*, which was shown to regulate galactose metabolism [22], with TCS07 responsive to glycoconjugated structures on proteins present on host cells [23]. The *RspA1* knock-out mutant, *ΔrspA1*, in *Streptomyces albus* demonstrated lower biomass accumulation and lower glucose consumption rates as compared to the parental strain A30 when cultivated in a defined minimal medium (MM) complemented with 75 mM glutamate [24]. The PolS-PolR TCS, identified in *Microlunatus phosphovorus* NM-1, was shown to regulate polyphosphate catabolism, and the response regulator PolR was found to directly bind to the promoters of genes involved in phosphate transport [25]. These examples outline the extent to which TCS regulation is present in bacterial metabolism.

In this study, EmaSR, a TCS participating in ethanol metabolism in *A. baumannnii*, was described for the first time. EmaR regulated ethanol metabolism via phosphorylation by EmaS. Few TCS have been reported to regulate ethanol metabolism to date, with only ExaS-ExaR and ElmS-GacA found to be indispensable for growth in ethanol of *Azoarcus* sp. strain BH72, an endophytic bacteria with nitrogen-fixing activity that colonizes rice roots under waterlogged conditions which favor the production of ethanol [26]. However, only one alcohol dehydrogenase (ExaA2) was regulated by ExaS-ExaR. *A. baumannii* also interacts with plants through colonization of rhizophores, and our previous study showed that *A. baumannii* possesses the ability to metabolize indole-3-acetic acid (IAA), a common phytohormone [3]. Previously, at least 7 genes in *A. baumannii* were annotated as alcohol dehydrogenases, and Adh4 was identified with high affinity toward ethanol, 1-propanol, and butanol [4]. Following up on this, in this study we were able to identify the EmaSR TCS, which has the ability to regulate genes involved in carbon metabolism (Figure 5B). However, transcriptomic analysis showed no significant differential gene expression of alcohol dehydrogenase, suggesting that either there may be different pathways to regulate alcohol dehydrogenase expression, or that alcohol dehydrogenase does not function in alcohol oxidation alone. Our observations suggest that the network for *A. baumannii* regulation of ethanol metabolism differs from that of *Azozrcus* sp. strain BH72 [26]. Interestingly, a complex TCS regulatory network for aerobic ethanol oxidation has been found and studied in *Pseudomonas aeruginosa* [27]. The network comprises 4 factors, in which ErdSR (ethanol metabolism regulatory factors) serve as initiators, with their signals further transferred to ErcSR or ErbSR for signal transduction [27]. ErdR was found to be highly conserved in *Pseudomonas* spp., and EmaR of *A. baumannii* shared 53.2% identity with ErdR of *P. putida* (Figure 1C). These findings suggest that there may be additional factors involved in *A. baumannii* ethanol metabolism. 

Ethanol metabolism is known to be important for stress resistance, but a previous study in *Pseudomonas savastanoi* suggests that it may be involved in virulence as well. The study found that a RhpSR TCS was able to regulate type-III secretion systems (T3SS) and alcohol dehydrogenase (*adhB*) in *P. savastanoi*, and nutrient-rich conditions enabled RhpR to directly regulate multiple metabolic pathways of *P. savastanoi*, while phosphorylation enabled RhpR to specifically control virulence and the bacterial cell envelope [28]. The EmaSR TCS in *A. baumannii* may be similarly involved in virulence mechanisms, and further studies are needed to confirm this.

In conclusion, this study identified a novel EmaSR TCS in *A. baumannii*, which was shown to regulate ethanol, acetate, and acetoin metabolism, and which was found to impact motility and biofilm formation. Further studies to examine the additional roles of the EmaSR TCS with regard to virulence and other aspects of metabolism and resistance are warranted.

## 4. Materials and Methods

### 4.1. Bacterial Strains, Plasmids, and Primers

*A. baumannii* ATCC 19606 and *Escherichia coli* strains were cultured in LB medium at 37◦C with agitation, and solid cultures were grown on LB medium containing 1.5% agar. Mutant *A. baumannii* strains were grown in M9 media (33.7 mM Na_2_HPO_4_, 22 mM KH_2_PO_4_, 8.55 mM NaCl, 9.35 mM NH_4_Cl, 1 mM MgSO_4_, 0.3 mM CaCl_2_), an MM to which 1% ethanol, or 5 mM citrate (5 mM citrate medium) was added according to experimental needs. The bacterial strains and plasmids used are presented in Table 1. The primers used in this study are presented in Table S4.

### 4.2. Marker-Less Mutation

Marker-less mutation was performed as previously described [4], with some minor modifications. Briefly, the adjacent regions of *emaS* and *emaR* intended for mutation were cloned into plasmid pk18*mobsacB* by Gibson assembly (New England Biolabs, Ipswich, MA, USA), and transformed into *E. coli* S17λπ to generate a donor strain for conjugation with *A. baumannii.* The *E. coli* donor strains and *A. baumannii* recipient strains were cultured in LB medium at 37 °C with shaking for 12 to 16 hours, after which a 200 μL aliquot of donor bacterial cells was mixed with recipient *A. baumannii* cells at a 1:20 ratio. The mixed cells were spun down and washed with 60 μL of conjugation buffer (1% NaCl, 10 mM MgSO_4_) to remove traces of LB medium, and the cell pellet was resuspended in 60 μL of conjugation buffer, then spotted onto a membrane filter (47 mm diameter, mixed cellulose esters, A020H047A; Advantech MFS, Dublin CA, USA) placed on 1.5% LB agar. After cultivation at 37 °C for 19 hours, filters were washed with conjugation buffer to remove bacterial cells, and spun down and resuspended in 200 μL of conjugation buffer, then plated to 1.5% LB agar with 50 μg/mL of ampicillin and 50 μg/mL of kanamycin. The first homologous recombination event enables the *E. coli* donor plasmid, which contains a kanamycin-resistant gene, to be integrated into the bacterial chromosome of *A. baumannii* recipient cells. Successful recombinants were cultured in LB medium containing 20% sucrose but without kanamycin, and as sucrose is lethal to bacterial cells expressing the *sacB* gene product, surviving recombinants are those that have excised the *sacB* gene in a second crossover event, thereby enabling the deletion of the target *emaS* and *emaR* genes. Deletion mutants were subsequently confirmed through PCR analysis.

### 4.3. Construction of Complement Plasmid and Strains

Complement strains were generated with a shuttle vector, pWH1266, which contains the replication origin for *E. coli* and *A. baumannii*. Promoters for the respective genes were amplified with primers as indicated in Table S4, and cloned into the *Eco*RI site of pWH1266 to respectively generate the complement plasmids, pS, pR, and pSR. For transformation, *A. baumannii* was cultured in LB broth overnight, and then subcultured in 50 mL fresh medium. Cells were collected after OD_600_ reached 0.6, and were washed twice with electroporation buffer (10% glycerol). Competent cells were resuspended in 30 mL of electroporation buffer and stored at −80 °C. At least 1000 ng of complement plasmid was mixed with 200 μL of competent cells and transferred into an electroporation cuvette (Hi-Lab Bioscience Co., Taichung, Taiwan), after which the Gene Pulser Xcell Microbial System (Bio-Rad, Taipei, Taiwan) was applied for electroporation at 1.8 kV. The resulting transformants were further checked by primers complementary with the sequences of pWH1266 (Figure S1). 

### 4.4. Recombinant EmaR and Antibody Production

Protein overexpression and purification methods were adapted from our previous study [14]. Briefly, the *emaR* gene was cloned into plasmid pQE80L (Qiagen, Hilden, Germany), which was then transformed into *E. coli* DH5α for production of recombinant protein. Ni-affinity chromatography was used for recombinant protein purification. Columns were charged with 1× charge buffer (5 mM NiSO4) for 50 minutes to enable Ni2+ binding with the column, after which columns were washed with 1× binding buffer (5 mM imidazole, 0.5 M NaCl, 20 mM Tris-HCl, pH = 7.9) for 50 minutes. Supernatant containing recombinant proteins was then passed through the column, following which the column was washed with 1× wash buffer (60 mM imidazole, 0.5 M NaCl, 20 mM Tris-HCl, pH = 7.9), and bound proteins were eluted with 1× elute buffer (1 M imidazole, 0.5 M NaCl, 20 mM Tris-HCl, pH = 7.9). The soluble fraction was collected and passed through Amicon Ultra-15 Centrifugal Filter-10 kDa units (Merck Millipore, Burlington, MA, USA) to eliminate proteins smaller than 10 kDA. Subsequently, at least 3 mg of total recombinant protein was prepared for polyclonal antibody preparation by Bio-Portech (Taipei, Taiwan). Polyclonal antibodies were applied for immunoblotting after Phos-tag^TM^ electrophoresis. 

### 4.5. Phos-tag^TM^ Analysis of EmaR

Wild-type and ΔS strains were cultured in LB broth with shaking at 37 °C overnight. Bacteria were subcultured in M9 medium containing 5 mM citrate with or without the presence of varying alcohols. Bacteria were collected after OD_600_ reached 0.6, and thencentrifuged and resuspended in 130 μL of 1M formic acid, 54 μL of sample buffer [130 mM Tris-Cl (pH 6.8), 6% SDS, 15% β-mercaptonethanol, 3% glycerol, 15% bromophenol blue] and 24 μL of 5N NaOH. Phos-tag^TM^ containing 12% SDS-acrylamide resolving gel was prepared with 50 μL of 50 mM Phos-tag^TM^, 1.25 ml of 1.5M Tris-Cl (pH8.8), 2.12 ml of 30% acrylamide solution, 50 μL of 10 mM MnCl_2_, 50 μL of 10% SDS, 1. 25 mL of ddH_2_O, 25 μL of 10% ammonium persulfate (APS), and 5 μL of N,N,N′,N′-tetramethylethylenediamine (TEMED). A 6% staking gel was prepared with 1 mL of 0.5M Tris-Cl (pH 6.8), 800 μL of 30% acrylamide solution, 40 μL of 10% SDS, 2.12 mL of ddH_2_O, 40 μL of 10% APS and 4 μL of TEMED. Electrophoresis of prepared samples was conducted at 120 V at room temperature for 90 minutes [29]. 

### 4.6. Motility

Motility assays were performed as previously described, with minor modifications [11]. Each strain was cultured in LB broth with agitation at 37 °C overnight. OD_600_ was determined, with OD_600_ equalized to 1 for each bacterium. A 2 μL sample of each strain was dropped on 0.5% LB agar, and plates were incubated at 37 °C, with images taken at 12-h intervals for at least 96 h. Swimming areas were analyzed by ImageJ (https://github.com/imagej/ImageJ, accessed on 16 October 2022).

### 4.7. Inorganic Stress Resistance

A single colony of each strain was inoculated in 3 mL of LB medium containing the corresponding antibiotics, and cultured for 12–16 hours at 37 °C with agitation. The overnight culture was refreshed with fresh medium to an OD_600_ of 0.1. Bacteria were then treated with 10 mM H_2_O_2_ for 20 minutes and 4% NaCl, after which viable counts were determined by dropping 5 μL of cultured medium on LB agar plates 6 times, and then calculating colony-forming units (CFU) after colonies appeared on the plates [4].

### 4.8. Biofilm Formation

Biofilm formation assays were performed as previously described, with minor modifications [3]. Bacteria were cultured in 3 ml of LB medium with the corresponding antibiotics for 12 hours, and then subcultured in LB medium containing different nutrient sources at an initial OD_600_ of 0.1. Cultures were grown in 96-well microtiter plates for 10 hours, after which crystal violet was added to each well at a final concentration of 0.1%, and cultures were maintained at room temperature for 30 minutes, following which the crystal violet-containing medium was removed, and wells were subsequently washed twice with distilled water. The stained biofilms in each well were dissolved in 300 μL of 70% ethanol, and incubated for 15 minutes at room temperature before OD_595_ analysis.

### 4.9. RNA Sequencing

RNA sequence reads generated from an Illumina HiSeq 2000 (San Diego, CA, USA) underwent selection, with reads containing adaptors, unknown sequences exceeding 10%, or low quality (more than half of all bases had quality of less than 5) removed. The clean reads were then mapped to the published *A. baumannii* ATCC 19606 genome (GenBank accession numbers: SRX3312085 and SRX3312086) using SOAP2 software [30]. Gene expression levels were calculated as reads per kilobase of genes per million reads (RPKMs) [31,32]. False discovery rates (FDR) [33] and the RPKM ratios of two samples were used to identify differentially expressed genes (DEGs). Genes with FDR ≤ 0.01 and an absolute log2 value ratio of greater than 1 were considered to be DEGs.

### 4.10. RNA Extraction and RT-PCR

Bacterial strains were cultured at 37 °C with agitation overnight, and then subcultured in 50 mL of M9 medium containing 5 mM citrate with or without 0.5% ethanol for at least 6 hours after OD_600_ reached 0.3. Samples were then collected and mixed with 0.1 volume of fixing solution (5% acid phenol, 95% ethanol). After centrifugation by 17,000× *g* at 4 °C, the supernatant was discarded, and cell pellets were stored at −80 °C for RNA extraction. Cell pellets were thawed on ice and resuspended in 1 mL of NucleoZOL (MACHEREY-NAGEL, Düren, Germany), then mixed thoroughly with 400 μL of diethyl pyrocarbonate (DEPC)-treated H_2_O, and incubated at room temperature for 15 minutes. The supernatant was recovered after centrifugation at 17,000× *g* at 4 °C for 20 minutes, then mixed with 5 μL of 100% 4-bromoanisole and incubated at room temperature for 10 minutes. Excess protein was then removed by centrifugation at 17,000× *g* at 4 °C for 20 minutes. The resulting RNA suspension was mixed with an equal volume of isopropanol for 15 minutes to induce RNA precipitation. The derived RNA pellet was washed twice with ice-cold 75% ethanol and resuspended in 30 μL of DEPC-treated H_2_O for analysis.

A Nanodrop 2000C spectrophotometer (Thermo Fisher Scientific, Waltham, MA, USA) was used to determine RNA concentrations, and a total of 2 μg of RNA was used to prepare cDNA. The RT-PCR mixture contained 10× reaction buffer, 200 U of MMLV high performance reverse transcriptase (Epicentre, Madison, WI, USA), 100 mM of dithiothreitol (DTT), 2.5 mM dNTP, and 1 nM of hexamer. The reaction was conducted in a Biometra TADVANCED Thermal Cycler (Analytik Jena, Jena, Germany). Gene-specific primers used to determine the presence and expression levels of the respective genes are listed in Table S4. The gyrase gene served as an internal control, and was amplified by PCR using the specific primers, gyrF and gyrR (Table S4).

### 4.11. Virulence Assay with G. mellonella

A virulence comparison was carried out with *A. baumannii* ATCC 19606 wild-type and *emaS, emaR,* and *emaSR* mutant strains. All procedures were performed as previously described, with minor modifications [14]. We selected 10 *G. mellonella* larvae with same total weight, and larvae were kept in petri dishes without food prior to infection. Overnight cultures of each strain were washed twice with PBS (0.137 M NaCl, 2.7 mM KCl, 10 mM Na_2_HPO_4_, 1.8 mM KH_2_PO_4_), then diluted in PBS. Larvae were subsequently infected with 5 × 10^6^ CFU of each strain, with bacteria in 10-µl aliquots injected into the hemocoel of each larva via the last left proleg using a Hamilton syringe. Infected larvae were then incubated at 37 °C, and scored for survival (alive/dead) every 24 hours, as well as for melanization over 96 hours, based on a previously described scoring method [34].

## Figures and Tables

**Table 1:** Plasmids and bacterial strains used in this study.

Plasmid	Description	Antibiotic Resistance (µg/mL)	Reference/Source
pK18*mobsacB*	Suicide vector for homologous recombination	Kan50	[15]
pK18D*emaS*	pK18*mobsacB* contains the upstream and downstream region of *emaS*	Kan50	This study
pK18D*emaR*	pK18*mobsacB* contains the upstream and downstream region of *emaR*	Kan50	This study
pWH1266	Ap^r^; Tc^r^; shuttle vector for *E. coli* and *A. baumannii* Ap^r^; fluorescent cytosolic biosensor	Amp50, Tc12.5	[16]
pS	pWH1266 contains P*_emaS_*- *emaS*	Amp50	This study
pR	pWH1266 contains P*_emaR_*- *emaR*	Amp50, Tc12.5	This study
pSR	pWH1266 contains P*_emaS_*- *emaS* and P*_emaR_*- *emaR*	Amp50	This study
pQE80L	Expression vector with *colE1* origin for His-tag fusion protein purification	Amp50	Qiagen
pQE80L-*emaR*	Ap^r^; *emaR* cloned into the *Bam*HI-*Sma*I site of pQE80L	Amp50	This study
**Strain**	**Description**		**Reference/Source**
*E. coli* DH5α	F^－^, *supE44*, *hsdR17*, *recA1*, *gyrA96*, *endA1*, *thi-1*, *relA1*, *deoR*, λ^－^		ATCC 53868
*Acinetobacter baumannii* ATCC 19606	Primary strain used in this study		[17]
Δ*emaS* (ΔS)	Marker-less *emaS* deletion mutant		This study
Δ*emaS*/pS (ΔS/S)	Δ*emaS* containing pS; Ap^r^		This study
Δ*emaS*/pR (ΔS/R)	Δ*emaS* containing pR; Ap^r^, Tc^r^		This study
Δ*emaR* (ΔR)	Marker-less *emaR* deletion mutant		This study
Δ*emaR*/pS (ΔR/S)	Δ*emaR* containing pS; Ap^r^		This study
Δ*emaR*/pR (ΔR/S)	Δ*emaR* containing pR; Ap^r^, Tc^r^		This study
Δ*emaSR* (ΔSR)	Marker-less *emaSR* double-deletion mutant		This study
Δ*emaSR*/pS (ΔSR/S)	Δ*emaSR* containing pS; Ap^r^		This study
Δ*emaSR*/pR (ΔSR/R)	Δ*emaSR* containing pR; Ap^r^, Tc^r^		This study
Δ*emaSR*/pSR (ΔSR/SR)	Δ*emaSR* containing pSR; Ap^r^		This study

Amp: ampicillin; Kan: kanamycin; Tc: tetracycline.

**Table 2:** EmaR binding boxes on EmaSR-regulated genes.

Name	Strand	Position *	*p*-Value	Sequence (5′-3′)
DJ41_568	+	−114 to −128	2.4 × 10^−6^	AAAAA ACTTATTTAAAACTT TTTAG
DJ41_1503	-	−107 to −121	1.5 × 10^−6^	TGTAA ATATTTGGAATAACT CAAAA
DJ41_2796	-	−107 to −121	1.1 × 10^−6^	TAAAA ATCATAAAAATAAGT TATAC
DJ41_3173	+	−150 to −164	2.3 × 10^−9^	TGAAA CCTTATGCCTATCAT AACCC
*emaR*	+	−15 to −29	1.6 × 10^−7^	GCCAT ACTTATGCTCAAGAT TACTT
*emaS*	-	−20 to −34	2.3 × 10^−9^	GGGTT ATGATAGGCATAAGG TTTCA

* Start codon (Met) as 0.

## Data Availability

The data presented in this study are available on request from the corresponding author. The data are not publicly available as the full dataset is undergoing analysis to guide future research and potential publication.

## Supplementary Materials

The following supporting information can be downloaded at: https://www.mdpi.com/article/10.3390/ijms232012606/s1.
