# Supplementary material for: Role of EmaSR in Ethanol Metabolism by Acinetobacter baumannii
Source: Int J Mol Sci. 2022 Oct 20;23(20):12606. doi: 10.3390/ijms232012606 (PMC9603970; doi:10.3390/ijms232012606)
Supplement: Supplementary file 1 [file ijms-23-12606-s001.zip › ijms-1974682-supplementary.pdf]

Role of EmaSR in Ethanol Metabolism by *Acinetobacter baumannii*

Supplementary Materials

Fig. S1A

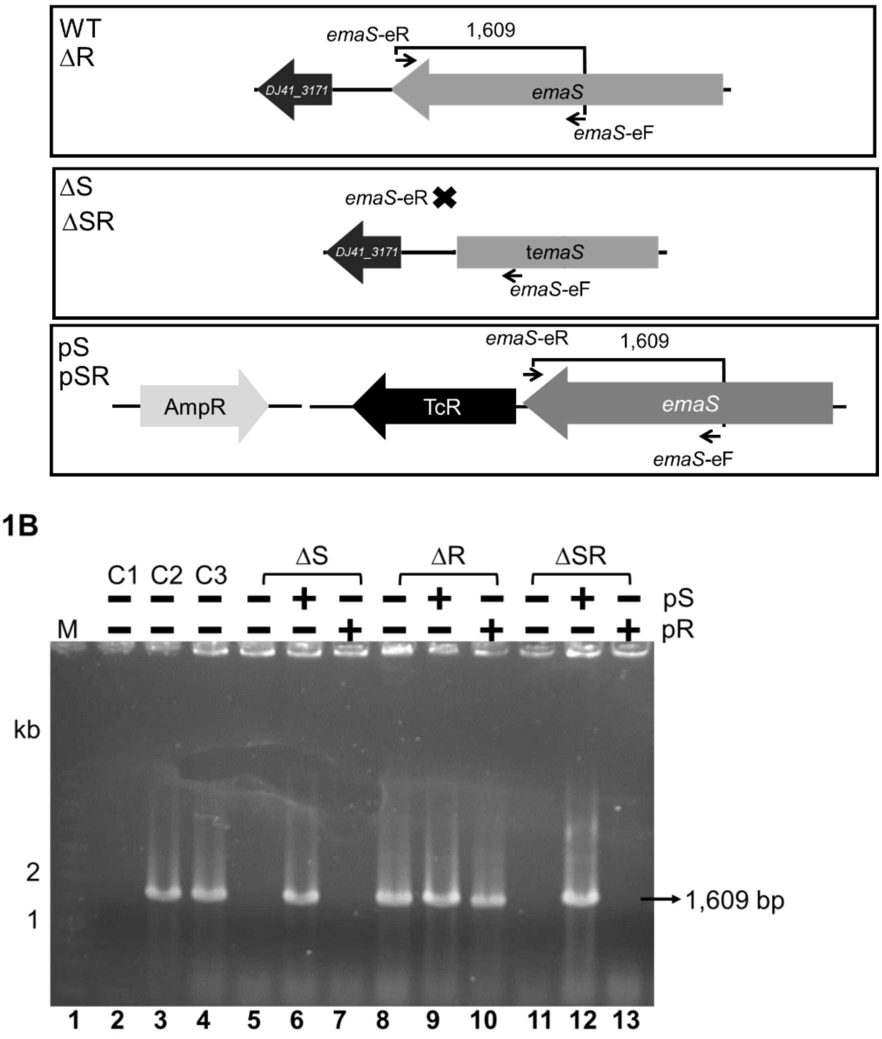

Figure S1. *EmaS* gene analysis of mutants and complementary strains. (**A**) Diagram of gene localization and primers for mutant and complementary strains. WT represents the wild-type strain. ΔR, ΔS, and ΔSR respectively represent the *emaR*, *emaS*, and *emaSR* mutants, while the pS and pSR strains respectively contain the complementation plasmids for *emaS* and *emaSR*. (**B**) PCR products analyzed by electrophoresis on a 0.8% agarose gel. The *emaS* gene was amplified by the emaS-eR and emaS-eF primers, with PCR fragment size of 1,069 bp. *EmaS* mutant strains have no PCR product. C1 is a negative control that used ddH_2O as a template, while C2 used the wild-type chromosomal DNA as a template, and C3 used pS in *E. coli* DH5α as a template. M represents the DNA size marker.

Fig. S2A

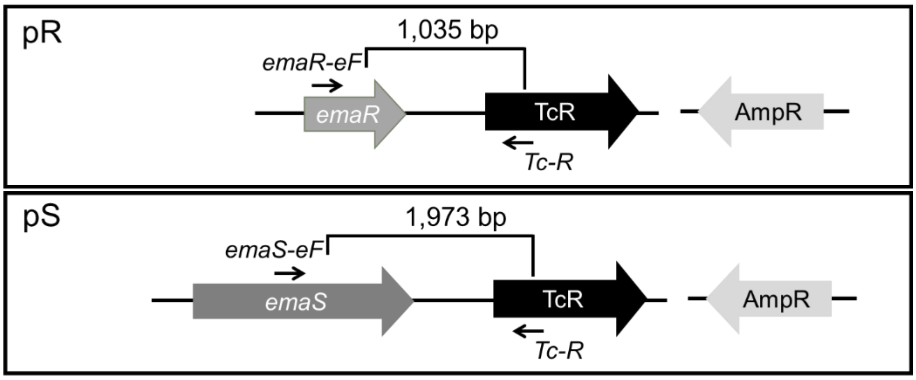

Fig. S2B

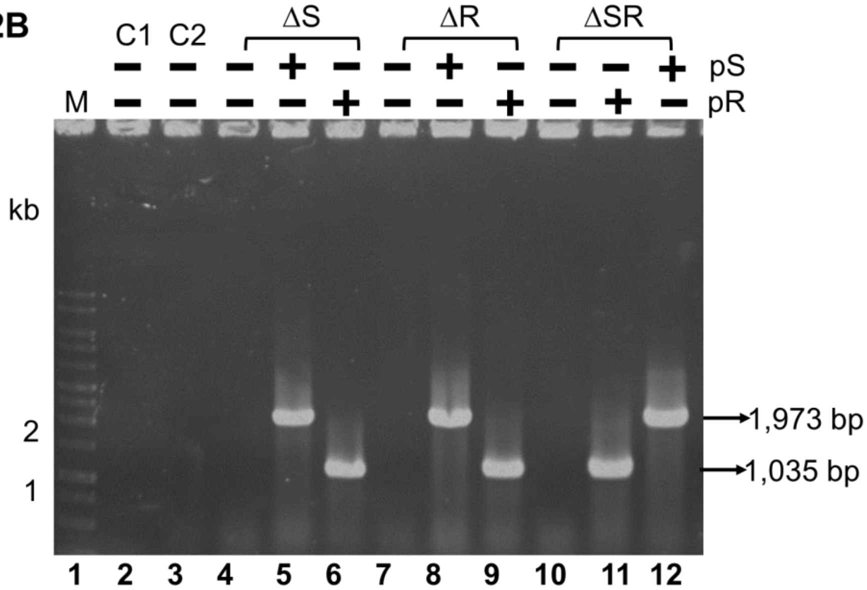

Figure S2. Complementary strains as confirmed by PCR. (**A**) Diagram of gene localization and primers for pR and pS, with pR confirmed using emaR-eF and Tc-R as primers, with expected amplification product of 1,035 bp, and pS confirmed using emaS-eF and Tc-R to amplify a DNA fragment of 1,973 bp in size. (**B**) PCR products analyzed by electrophoresis on a 0.8% agarose gel. C1 is a negative control that used ddH_2O as a template, while C2 used the wild-type strain without complementary plasmid as a template.

Table S1. Differentially regulated genes in the Δ*emaSR* mutant strain versus the ATCC 19606 wild-type strain in key functional groups.

Category and locus tag	Gene	Log_2 fold change[a]	p-value	Predicted function
**Carbon metabolism**				
DJ41_2796	-	6.21	3.53×10^{-9}	succinate CoA transferase family protein
DJ41_569	-	5.78	2.12×10^{-8}	E3 binding domain protein
DJ41_568	*lpdA*	4.01	1.82×10^{-5}	dihydrolipoyl dehydrogenase
DJ41_2528	-	3.33	0.00019	isocitrate lyase
DJ41_1153	*pta*	2.34	0.00574	phosphate acetyltransferase
DJ41_1152	*ackA*	2.23	0.00789	acetate kinase
DJ41_3568	*gltA*	1.66	0.03356	citrate (Si)-synthase
DJ41_227	*fumC*	1.61	0.03873	fumarate hydratase, class II
**Phenylalanine metabolism**				
DJ41_3060	*hppD*	3.51	0.00012	4-hydroxyphenylpyruvate dioxygenase
DJ41_2050	*paaJ*	2.43	0.00461	phenylacetate-CoA oxygenase, PaaJ subunit
DJ41_2049	-	2.08	0.01162	2Fe-2S iron-sulfur cluster binding domain protein
DJ41_2052	-	1.96	0.02361	phenylacetic acid degradation B family protein
DJ41_3262	-	1.94	0.01876	pyridine nucleotide-disulfide oxidoreductase family protein
DJ41_2053	*paaA*	1.90	0.01821	phenylacetic acid degradation protein paaA
DJ41_2051	*paaC*	1.84	0.02349	phenylacetic acid degradation protein paaC
DJ41_2054	*paaN*	1.73	0.02970	phenylacetic acid degradation protein paaN
DJ41_2048	-	1.61	0.04592	enoyl-CoA hydratase/isomerase family protein
**Biosynthesis of cofactors**				
DJ41_568	*lpdA*	4.01	1.82×10^{-5}	dihydrolipoyl dehydrogenase
DJ41_3060	*hppD*	3.51	0.00012	4-hydroxyphenylpyruvate dioxygenase
**Biosynthesis of siderophore group nonribosomal peptides**				
DJ41_1728	-	3.75	8.51×10^{-5}	isochorismate synthase family protein
DJ41_1721	*entB*	2.59	0.00266	isochorismatase
DJ41_512	-	2.55	0.005	short chain dehydrogenase family protein
DJ41_1720	-	2.25	0.00716	(2,3-dihydroxybenzoyl)adenylate synthase
**Purine metabolism**				
DJ41_2077	-	-4.36	8.52×10^{-5}	cytidine and deoxycytidylate deaminase zinc-binding region family protein
**Tyrosine metabolism**				
DJ41_3063	*maiA*	3.85	8.99×10^{-5}	maleylacetoacetate isomerase
DJ41_3064	*fahA*	3.53	0.00013	fumarylacetoacetase
DJ41_3060	*hppD*	3.51	0.00012	4-hydroxyphenylpyruvate dioxygenase
**Two-component system**				

DJ41_2555	-	2.35	0.00479	TonB-dependent siderophore receptor family protein
**Sulfur metabolism**				
DJ41_1587	-	-2.11	0.03619	sulfate ABC transporter, sulfate-binding family protein
DJ41_3169	*msuE*	-2.31	0.02509	FMN reductase

[a] Log_2 fold change = Log_2(WT/Δ*emaSR*)

Table S2. Differentially regulated genes in the Δ*emaR* mutant strain versus the ATCC 19606 wild-type strain in key functional groups.

Category and locus tag	Gene	Log_2 fold change[a]	p-value	Predicted function
**Carbon metabolism**				
DJ41_2796	-	7.03	2.93×10^{-10}	succinate CoA transferase family protein
DJ41_569	-	4.88	1.37×10^{-6}	e3 binding domain protein
DJ41_568	*lpdA*	4.64	3.77×10^{-6}	dihydrolipoyl dehydrogenase
DJ41_2528	-	2.82	0.00199	isocitrate lyase
DJ41_1153	*pta*	2.75	0.00304	phosphate acetyltransferase
DJ41_1152	*ackA*	2.68	0.00371	acetate kinase
**Phenylalanine metabolism**				
DJ41_3262	-	1.97	0.0293	pyridine nucleotide-disulfide oxidoreductase family protein
**Biosynthesis of cofactors**				
DJ41_3276	-	4.72	0.00084	hypothetical protein
DJ41_568	*lpdA*	4.64	3.77×10^{-6}	dihydrolipoyl dehydrogenase
DJ41_3509	*nqO2*	3.86	0.01529	ribosyldihydronicotinamide dehydrogenase (quinone)
**Biosynthesis of siderophore group nonribosomal peptides**				
DJ41_1728	-	2.17	0.01904	isochorismate synthase family protein
**Purine metabolism**				
DJ41_3122	*uraD*	3.95	0.03722	OHCU decarboxylase
DJ41_3118	-	2.96	0.01487	FAD binding domain protein
DJ41_3123	-	2.66	0.04244	polysaccharide deacetylase family protein
DJ41_2077	-	-3.83	0.00025	cytidine and deoxycytidylate deaminase zinc-binding region family protein
**Two-component system**				
DJ41_3249	-	16.78	0.00028	autoinducer synthetase family protein
DJ41_3468	-	2.29	0.01665	cheW-like domain protein
DJ41_1407	-	2.17	0.04247	PAS fold family protein
**Bacterial secretion system**				
DJ41_1170	*tatC*	4.47	0.00751	twin arginine-targeting protein translocase TatC

[a] Log_2 fold change = Log_2(WT/Δ*emaR*)

Table S3. Differentially regulated genes in the Δ*emaS* mutant strain versus the ATCC 19606 wild-type strain in key functional groups.

Category and locus tag	Gene	Log_2 fold change[a]	p-value	Predicted function
**Carbon metabolism**				
DJ41_2796	-	6.66	6.88×10^{-10}	succinate CoA transferase family protein
DJ41_2528	-	2.65	0.00208	isocitrate lyase
DJ41_1152	*ackA*	2.47	0.00443	acetate kinase
DJ41_1153	*pta*	2.47	0.00433	phosphate acetyltransferase
DJ41_227	*fumC*	1.73	0.03169	fumarate hydratase, class II
DJ41_1959	-	-2.15	0.03472	catalase family protein
DJ41_2119	-	-2.67	0.00812	D-ala D-ala ligase family protein
DJ41_568	*lpdA*	-2.91	0.00352	dihydrolipoyl dehydrogenase
DJ41_569	-	-3.05	0.00219	e3 binding domain protein
**Phenylalanine metabolism**				
DJ41_3060	*hppD*	3.21	0.00037	4-hydroxyphenylpyruvate dioxygenase
DJ41_3262	-	2.51	0.00415	pyridine nucleotide-disulfide oxidoreductase family protein
DJ41_2051	*paaC*	2.03	0.01538	phenylacetic acid degradation protein paaC
DJ41_2054	*paaN*	2.00	0.01565	phenylacetic acid degradation protein paaN
DJ41_2050	*paaJ*	1.97	0.01879	phenylacetate-CoA oxygenase, PaaJ subunit
DJ41_2052	-	1.73	0.04316	phenylacetic acid degradation B family protein
DJ41_2053	*paaA*	1.57	0.04764	phenylacetic acid degradation protein paaA
**Biosynthesis of cofactors**				
DJ41_3509	*nqO2*	3.28	0.02682	ribosyldihydronicotinamide dehydrogenase quinone
DJ41_3060	*hppD*	3.21	0.00037	4-hydroxyphenylpyruvate dioxygenase
DJ41_3276	-	3.06	0.01392	hypothetical protein
DJ41_572	*lipA*	-2.65	0.01033	lipoyl synthase
DJ41_568	*lpdA*	-2.91	0.00352	dihydrolipoyl dehydrogenase
**Biosynthesis of siderophore group nonribosomal peptides**				
DJ41_1728	-	4.34	1.29×10^{-5}	isochorismate synthase family protein
DJ41_1721	*entB*	3.80	5.65×10^{-5}	isochorismatase
DJ41_1720	-	3.51	0.00014	(2,3-dihydroxybenzoyl)adenylate synthase
DJ41_512	-	3.44	0.00041	short chain dehydrogenase family protein
**Purine metabolism**				
DJ41_3122	*uraD*	3.52	0.04837	OHCU decarboxylase
DJ41_3123	-	2.84	0.02402	polysaccharide deacetylase family protein
DJ41_2077	-	-4.56	4.28×10^{-5}	cytidine and deoxycytidylate deaminase zinc-binding region family protein

**Tyrosine metabolism**				
DJ41_3063	*maiA*	3.95	7.4×10^{-5}	maleylacetoacetate isomerase
DJ41_3064	*fahA*	3.58	0.00012	fumarylacetoacetase
**Two-component system**				
DJ41_3249	-	3.85	0.03103	autoinducer synthetase family protein
DJ41_2555	-	3.54	0.00010	TonB-dependent siderophore receptor family protein
DJ41_3060	*hppD*	3.21	0.00037	4-hydroxyphenylpyruvate dioxygenase
DJ41_3468	-	1.84	0.03365	cheW-like domain protein
**Bacterial secretion system**				
DJ41_1170	*tatC*	4.13	0.00921	twin arginine-targeting protein translocase TatC
DJ41_1169	*tatB*	3.81	0.04078	twin arginine-targeting protein translocase TatB

Table S4. List of plasmids used in this study.

Name	Sequence (5′-3′)	Function
*emaS*upF	ACATGACCCAAGGGGTAAGCTAATCATTTATCGCTTCCC	Construction of *emaS* mutant
pK18_*emaS*upR	GAGCTCGGTACCCGGGAATCAACAAAGAGAGCTGCG	Construction of *emaS* mutant
pK18_*emaS*downF	ACGACGGCCAGTGCCATGCATCAACAGGTATGTTAC	Construction of *emaS* mutant
*emaS*downR	TGGGAAGCGATAAATGATTAGCTTACCCCTTGGGTCATGT	Construction of *emaS* mutant
*emaR*upF	TTCATGAAACAGCTTCGGTTATGTTCCACTTAGGCAAGGC	Construction of *emaR* mutant
pK18_*emaR*upR	GAGCTCGGTACCCGGGTCACCTTATACGACTTTAGT	Construction of *emaR* mutant
pK18_*emaR*downF	ACGACGGCCAGTGCCATAACTGTTCACTTTTCACCG	Construction of *emaR* mutant
*emaR*downR	GCCTTGCCTAAGTGGAACATAACCGAAGCTGTTTCATGAA	Construction of *emaR* mutant
emaS-eF	*Bam*HI GGATCCGCTTTAGGACAAGTGGCTAA	Expression of *emaS*
emaS-eR	*Hind*III AAGCTTTTAGTCTGTCGTGCTGGCAA	Expression of *emaS*
emaR-eF	*Bam*HI TTGGATCCATGGAAAATGGGGCAGAACCTGA	Expression of *emaR*
emaR-eR	*Sma*I TTCCCGGGTTAGATTTTTTTATCTTCAA	Expression of *emaR*
pWH-*emaS*cF	GACAGCTTATCATCGATAAACCAAACTCCTTACATAGTG	Complement of *emaS*
pWH-*emaS*cR	GATAAACTACCCCATTAATTAGTCTGTCGTGCTGGCAA	Complement of *emaS*
pWH-*emaR*cF	CCCTTTCGTCTTCAAGGATTTGGTTCCTAAGCCTAA	Complement of *emaR*
pWH-*emaR*cR	AGCTGTCAAACATGAGTTAGATTTTTTTATCTTCAA	Complement of *emaR*
DJ41_571-rF	TGCAATTAACGGAAGAGCAA	RT-PCR
DJ41_571-rR	TGTCCACGGTGTGTTGAAGT	RT-PCR
DJ41_2796-rF	TGGAATGACCGTTGGTATGA	RT-PCR
DJ41_2796-rR	TAAACATGACTTCGCCGTTG	RT-PCR
DJ41_3170-rF	AATCGTTGATGATCATCCTT	RT-PCR
DJ41_3170-rR	TCTTCGTGTGCAGAAACCAC	RT-PCR
DJ41_3172-rF	CCTCACCCTCAACCCAAATA	RT-PCR
DJ41_3172-rR	CTTACCCCTTGGGTCATGTG	RT-PCR
DJ41_3174-rF	TATTACCAAATGGGCGGCTA	RT-PCR
DJ41_3174-rR	CCCGAGTGAATAGAGCAAGC	RT-PCR
DJ41_3688-rF	GGCGGTTTATCTGAGTTTGT	RT-PCR
DJ41_3688-rR	TTTGTGGAATGTTGTTTGTG	RT-PCR
DJ41_3771-rF	AAGGGCAACTCTCGTCTCAA	RT-PCR
DJ41_3771-rR	CAGGCGCAAATACATCAAAA	RT-PCR
DJ41_571-rF	TGCAATTAACGGAAGAGCAA	RT-PCR

* Start codon (Met) as 0.
